# Yield of non-invasive imaging in MRI-negative focal epilepsy

**DOI:** 10.1007/s00415-023-11987-6

**Published:** 2023-11-01

**Authors:** Christian Czarnetzki, Laurent Spinelli, Hans-Jürgen Huppertz, Karl Schaller, Shahan Momjian, Johannes Lobrinus, Maria-Isabel Vargas, Valentina Garibotto, Serge Vulliemoz, Margitta Seeck

**Affiliations:** 1 EEG & Epilepsy Unit, Department of Clinical Neurosciences, University Hospital and Faculty of Medicine, University of Geneva, 4, Rue Gabrielle-Perret-Gentil, 1211 Geneva, Switzerland; 2 Department of Clinical Neurosciences, Neurosurgery Clinic, University Hospital of Geneva, Geneva, Switzerland; 3 Swiss Epilepsy Clinic, Klinik Lengg, Zurich, Switzerland; 4 Department of Clinical Pathology, Faculty of Medicine, University Hospital of Geneva, Geneva, Switzerland; 5 Department of Radiology, Faculty of Medicine, University Hospital of Geneva, Geneva, Switzerland

**Keywords:** MRI-negative, Epilepsy surgery, Focal epilepsy, Adults, Children, Non-lesional, PET, SPECT, SISCOM, ESI, Morphometry

## Abstract

**Objective:**

The absence of MRI-lesion reduces considerably the probability of having an excellent outcome (International League Against Epilepsies [ILAE] class I–II) after epilepsy surgery. Surgical success in magnetic-resonance imaging (MRI)-negative cases relies therefore mainly on non-invasive techniques such as positron-emission tomography (PET), subtraction ictal/inter-ictal single-photon-emission-computed-tomography co-registered to MRI (SISCOM), electric source imaging (ESI) and morphometric MRI analysis (MAP). We were interested in identifying the optimal imaging technique or combination to achieve post-operative class I-II in patients with MRI-negative focal epilepsy.

**Methods:**

We identified 168 epileptic patients without MRI lesion. Thirty-three (19.6%) were diagnosed with unifocal epilepsy, underwent surgical resection and follow-up ⩾ 2 years. Sensitivity, specificity, predictive values, and diagnostic odds ratio (OR) were calculated for each technique individually and in combination (after co-registration).

**Results:**

23/33 (70%) were free of disabling seizures (75.0% with temporal and 61.5% extratemporal lobe epilepsy). None of the individual modalities presented an OR > 1.5, except ESI if only patients with interictal epileptiform discharges (IEDs) were considered (OR 3.2). On a dual combination, SISCOM with ESI presented the highest outcome (OR = 6). MAP contributed to detecting indistinguishable focal cortical dysplasia in particular in extratemporal epilepsies with a sensitivity of 75%. Concordance of PET, ESI on interictal epileptic discharges, and SISCOM was associated with the highest chance for post-operative seizure control (OR = 11).

**Conclusion:**

If MRI is negative, the chances to benefit from epilepsy surgery are almost as high as in lesional epilepsy, provided that multiple established non-invasive imaging tools are rigorously applied and co-registered together.

**Supplementary Information:**

The online version contains supplementary material available at 10.1007/s00415-023-11987-6.

## Introduction

Epilepsy surgery is an important therapeutic option, which should be offered whenever possible for patients with pharmacoresistant epilepsy. Comprehensive non-invasive imaging is key to obtaining excellent surgical results. Ictal and interictal scalp electroencephalography (EEG) monitoring and magnetic resonance imaging (MRI) are the most important tools: chances of post-operative seizure control are highest if a lesion is detected, concordant with EEG and seizure semiology, and resected completely [1]. In previous meta-analysis and retrospective studies, the percentage of patients with a good outcome and with MRI-negative epilepsy (MNE) is reported to be as low as 30–50% [1, 2]. Numbers are lowest for patients with MRI-negative extratemporal lobe epilepsy with 38–46% of patients [3–6] compared to MRI-negative temporal epilepsy, with post-operative seizure control in 55–76% of cases [7–11].

Since it is well-established knowledge that MRI-negative epilepsy is associated with a markedly lower surgical success, there is a certain reluctance to offer surgical therapy (and presurgical evaluation). However, we hypothesize that the outcome in MRI-negative epilepsy is better than reported in most previous studies if supplementary imaging tools are rigorously used together, such as positron-emission-tomography (PET), single-photon emission computed tomography (SPECT), electric source imaging (ESI) based on IEDs in the EEG, and MR-based analysis techniques like morphometric MRI analysis (MAP). Most studies in MRI-negative focal epilepsy investigate the yield of a certain technique, e.g., PET [12, 13] or SISCOM [14, 15], and more recently the yield of multimodal imaging [16]. Here, we present our experience on MRI-negative patients using PET, ESI, MAP and whenever possible SISCOM and determine which technique or combination has the largest impact on post-operative seizure control.

## Methods

### Patient population

We enrolled all patients assessed for pharmacoresistant epilepsy at the University Hospitals of Geneva between 2000 and 2018 and screened them according to the following inclusion criteria: (a) unifocal epilepsy as suggested by the EEG and semiology, (b) absence of MRI lesion, (c) underwent resective surgery, (d) follow-up of at least 2 years. The exclusion criteria were: (a) diffuse or multifocal epilepsy, (b) palliative surgery, (c) follow-up < 2 years, (d) genetic or auto-immune origin of their epilepsy. The definition of genetic or auto-immune epilepsy is the same as mentioned in [17]. All patients received video-EEG monitoring and 1.5 or 3T MRI. “MRI-negative” or “non-lesional” refers to the absence of an identifiable lesion despite the use of dedicated epilepsy MRI protocols as determined by an experienced neuroradiologist with a special interest in epilepsy. The MRI protocol included specific sequences with axial and coronal FLAIR and T2/STIR, axial hemosiderin/calcification-sensitive sequences, and 3D-T1 as proposed by [18].

**Fig. 1:**
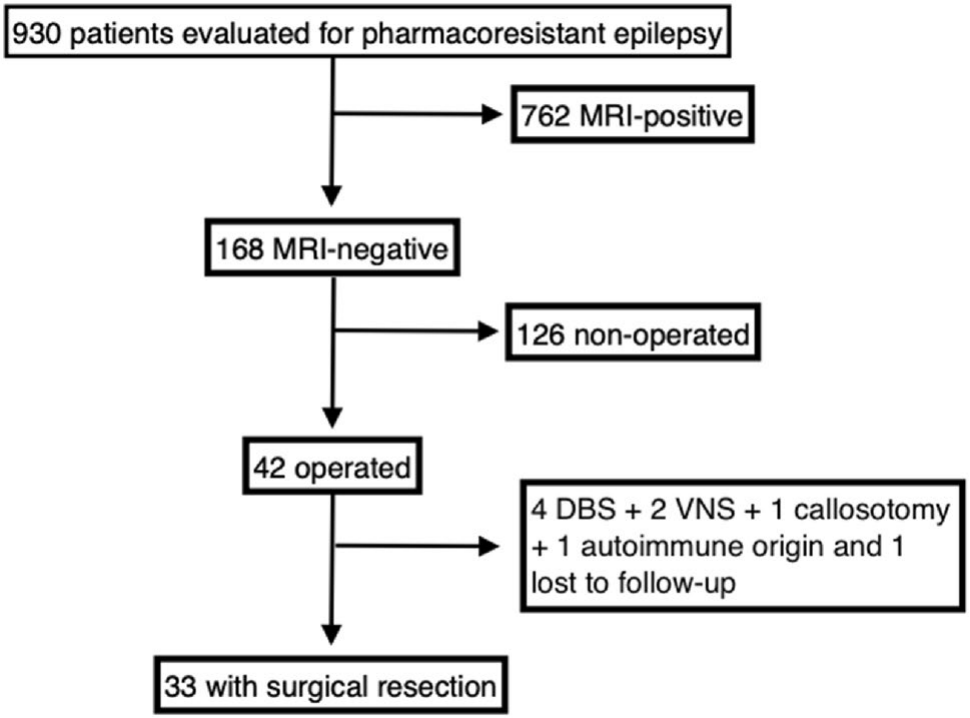
Surgical resection in epileptic patients with MRI-negative results

Of the 930 patients evaluated at our hospital for pharmacoresistant epilepsy, 168 were found to be MRI-negative and 42 underwent surgery. Not all patients identified as MRI-negative were operated, as some did not want further investigations, had epilepsy of multifocal or generalized origin, presented a good response to drug treatment after adaptation, or suffered from non-epileptic seizures. Seven of these 42 had only functional surgery (vagal nerve stimulation, deep brain stimulation, and callosotomy), one presented multifocal seizures after surgery and later diagnosed with an autoimmune origin of their epilepsy and another patient was lost to follow-up. Hence, 33 fulfilled our inclusion criteria and were subject to analysis (see Fig. 1).

All patients received PET, ESI, and ictal SPECT whenever possible; MAP since 2012. All four imaging could be obtained in 24 patients, and three in 9 of them. In our institution, ictal SPECT imaging is offered during working days between 8 am and 4 pm. However, despite the availability of the tracer for five working days, ictal SPECT could not be obtained in all patients. 28/33 underwent also intracranial EEG (iEEG) recordings before surgery (depth and/or subdural electrodes). Intracranial electrode implantation was guided by the results of non-invasive exams and was carried out in order to confirm the epileptic zone (EZ) and/or delineate the epileptogenic focus from the eloquent cortex.

The Regional Research Ethics Committee (CCER) approved this retrospective study.

### Positron emission tomography (PET)

PET was performed using 2-[18 F]fluoro-2-deoxy-d-glucose (FDG) during the interictal phase (at least 24 h after the last seizure). In-house monitoring 24 h before and during the PET is carried out to monitor for subclinical or amnestic seizures, leading to incorrect hyper- or isometabolism. After fasting for at least 6 h, patients received an injection of 200–250 MBq of [18 F]-FD. In children, the dose was adapted to the weight. Patients rested in a quiet and dimly lit room, with continuous EEG monitoring to exclude subclinical seizures during tracer uptake, which would interfere with the proper interpretation of the exam. PET images were acquired 30 min after injection on the following tomographs over the years: ECAT ART (CTI, Knoxville, US), Siemens Biograph Hi-Rez, TruePoint, mCT, and Vision (Siemens, Erlangen, Germany). Scanning duration was 20–25 min. One expert reader (VG) who was blind regarding the possible epileptogenic zone analyzed all PET images visually. All images were reanalyzed after coregistration of the PET images to the patient’s individual MRI images. Statistical parametric mapping (SPM) was performed (SPM8; Wellcome Department of Cognitive Neurology, London, UK) and data sets from each patient were compared to a reference set of brain PET scans from 38 young healthy controls (20 men; mean age 35 years, range 18–53 years).

### Single-photon emission computed tomography (SPECT)

Ictal SPECT scans were performed by administering 740 MBq of stabilized-hexamethylpropylene-amino-oxime (HMPAO) or ethyl-cysteinate dimer (ECD) labeled with 99mTc for adults and adapted doses to body weight in case of children. Scans were acquired 30–120 min after the radioisotope injection using a three-head (Toshiba CGA-9300, Tokyo, Japan) or two-head (Symbia, Siemens, Erlangen, Germany) gamma camera. Subtraction ictal SPECT from the interictal SPECT, co-registered with MRI (SISCOM) was carried out and the maximal area of hyperperfusion was identified. Interictal SPECT was obtained after > 24–48 h without seizures.

### Electrical source imaging (ESI)

Electric source imaging (ESI) is a method based on the reconstruction of brain activity in the 3D space based on scalp electrodes as described elsewhere [19]. In the present context, the source of the patient’s interictal discharges is reconstructed within the patient’s own MRI using the cerebral grey matter as solution space. ESI is obtained with high-density recordings (> 64 EEG channels; PhilippsNeuro, Eugene, OR, USA) of 2–12 h duration with in-house analysis. Since 5 years, ESI is performed as a semi-automated procedure obtained from the entire video-EEG recording period (on average: 9 days) using 37 scalp electrodes (Epilog©, Belgium) with comparable accuracy results compared to shorter high-density ESI with 128–256 electrodes [20]. The absence of interictal epileptiform discharges was considered a false negative. We also conducted a separate analysis for those patients with interictal epileptic discharges in order to provide prognostic data also for those with a clearly identifiable EEG focus.

### Morphometric MRI analysis (MAP)

Morphometric analysis was performed by means of the Morphometric Analysis Program (MAP; *version 2018*). Using algorithms of the SPM 12 software (http://www.fil.ion.ucl.ac.uk/spm/) running in MATLAB R2020b (MathWorks, Natick, MA, U.S.A) native T1-weighted 3D MRI images were segmented into gray matter, white matter, and cerebrospinal fluid maps, and normalized to the Montreal Neurological Institute (MNI) space. Building on this, MAP, as described in detail elsewhere [21, 22], was employed to create morphometric maps that highlight MRI hallmarks of focal cortical dysplasia (i.e., abnormal extension of gray matter in white matter in case of abnormally deep sulci, abnormal thickening of the cortical ribbon, and blurring of the gray-white matter junction). For MAP analysis, extension, junction, and Thickness image maps were considered. The “combined map” represents the maximum *z* score of the three maps for each voxel and is verified by visual analysis.

This technique was not available at the onset of our surgical program, which is why not all patients underwent supplementary MRI analysis.

### Coregistration

All modalities were coregistered to the T1 MRI for each patient. The images were uploaded and computed in Analyze 9.0 (AnalyzeDirect, Overland Park, Kansas, USA), by setting the level of significance to 2 standard deviations.

### Statistical analysis

Each method was analyzed with respect to the superposition of the area of maximal anomaly or of the maximum ESI with the resected region. The test was (1) “concordant” if it depicted an area which was localized within or mostly within (> 80%) the resected region (determined visually for ESI, morphometry, and SPECT, statistical maps for PET); (2) “discordant” if the identified localization was outside or mostly outside of the resected region; (3) “non-contributing” if the test was considered as normal or multifocal, i.e., not contributing to decision making regarding focus localization and consecutive surgery. We compared concordant with all other exams (i.e., discordant and non-contributing).

We defined a patient as having a good outcome when there were no disabling seizures, i.e., with alteration of consciousness during a follow-up of at least 2 years.

We calculated specificity, sensitivity, positive predictive (PPV), and negative predictive values (NPV) as well as odds ratio (OR) for each imaging method comparing patients with a good outcome (ILAE 1–2) to the patients with ILAE 3–6 outcome. We defined specificity as the percentage of patients who presented discordant or non-contributing results and were scored with an ILAE class 3 or lower, and sensitivity as the % of patients who showed concordant findings with respect to the resection site and presented an ILAE class 1–2.

## Results

### Patient characteristics

Clinical patient characteristics are found in Table 1 and individual data of the 33 patients are summarized in Table 2 (supplementary material).

**Table 1:** Summary of the clinical patient characteristics

Clinical characteristics	
Gender ratio (M/F): 15/18 Mean age at onset (years): 11.5 ± 8.0 Mean age at operation (years): 27.2 ± 13.1 Patients with extratemporal/temporal epilepsy: 13/20 Mean post-op follow-up period (months): 60.5 ± 63.0 Post-operative outcome: ILAE 1–2: 23 ILAE 3: 2 ILAE 4: 1 ILAE 5: 6 ILAE 6: 1 Intracranial recordings: 28 (85%)	

Twenty-two patients (67%) were adults and 11 (33%) were younger than 18 years at operation. The outcome was classified according to the ILAE classification of epilepsy surgery as seen in [23]. Overall, 23 (69.7%) had an ILAE 1–2 outcome, i.e., were completely seizure-free, or had only auras. Ten patients (30.3%) showed moderate or no improvement postoperatively (ILAE class 3 or lower). Twenty patients (61%) were diagnosed with non-lesional temporal lobe epilepsy (TLE), thirteen (39%) with non-lesional extratemporal lobe epilepsy (ETLE). 75% (15/20) of TLE and 61.5% (8/13) of ETLE patients had no more seizures with impairment of consciousness (*p* = 0.41).

All patients underwent PET imaging, 23 had electric source imaging (ESI) using IEDs, and in 30 patients we could obtain an ictal single photon emission tomography (SPECT). In 27 patients, morphometric MRI analysis (MAP) was carried out.

### Results of imaging

The results of individual and combined exams are found in Table 2.

**Table 2:** Sensitivity, specificity, PPV and NPV for each method

*N* total = 33	Sensitivity	Specificity	PPV	NPV	Accuracy	OR
PET (*N* = 33)	13/18 (72%)	5/15 (33%)	13/23 (57%)	5/10 (50%)	53%	1.3
ESI (*N* = 33)	12/17 (71%)	5/16 (31%)	12/23 (52%)	5/10 (50%)	51%	1.1
ESI (spikes only; *N* = 23)	12/17 (71%)	4/6 (67%)	12/14 (86%)	4/9 (44%)	63%	3.2
SISCOM (*N* = 30)	15/19 (79%)	3/11 (27%)	15/23 (65%)	3/7 (43%)	53%	1.4
MAP (*N* = 27)	3/4 (75%)	7/23 (30%)	3/19 (16%)	7/8 (88%)	53%	1.3
Combination of 2 exams						
PET + SISCOM (*N* = 30)	9/11 (82%)	5/19 (26%)	9/23 (39%)	5/7 (71%)	54%	1.6
PET + ESI (*N* = 33)	7/9 (78%)	8/24 (33%)	7/23 (30%)	8/10 (80%)	56%	1.75
SISCOM + MAP (*N* = 24)	2/3 (67%)	6/21 (29%)	2/17 (12%)	6/7 (86%)	48%	0.5
SISCOM + ESI (*N* = 30)	10/12 (83%)	7/18 (39%)	10/21 (48%)	7/9 (78%)	61%	1.5
PET + MAP (*N* = 27)	1/1 (100%)	8/26 (31%)	1/19 (5%)	8/8 (100%)	55%	1.4
ESI + MAP (*N* = 27)	1/ 2 (50%)	7/25 (28%)	1/19 (5%)	7/8 (88%)	39%	0.4
Combinations with ESI including patients with spikes only						
PET + ESI (*N* = 23)	7/9 (78%)	8/14 (57%)	7/13 (54%)	8/10 (80%)	67%	4.7
MAP + ESI (*N* = 27)	1 /2 (50%)	8/16 (50%)	1/9 (11%)	8/9 (89%)	50%	1.0
SISCOM + ESI (*N* = 21)	9/12 (75%)	6/9 (67%)	9/12 (75%)	6/9 (67%)	71%	6.0
SISCOM + ESI + PET (*N* = 21)	5/5 (100%)	8/16 (50%)	5/13 (38%)	8/8 (100%)	75%	11

Please note that that the largest OR for a good outcome is obtained with SISCOM + ESI + PET. None of the patients had a concordance of all 4 imaging tools, thus this combination was not included in the present analysis

*PET* positron-emission tomography, *SISCOM* subtraction ictal single-photon emission CT coregistered to MRI, *ESI* electric source imaging, *MAP* morphometric MRI-analysis. *PPV* positive predictive value, *NPV* negative predictive value.

A subgroup analysis of individual exams in patients with temporal and extra-temporal epilepsy was performed and is found in Table 3.

**Table 3:** Comparison of yields of imaging tools in patients with temporal lobe and extra-temporal lobe epilepsy

	Sensitivity	Specificity	PPV	NPV	OR
PET (*N* = 33)					
Temporal (*N* = 20)	10/14 (71%)	1/6 (17%)	10/15 (67%)	1/5 (20%)	0.5
Extra-temporal (*N* = 13)	3/4 (75%)	4/9 (44%)	3/8 (38%)	4/5 (80%)	2.4
SISCOM (*N* = 30)					
Temporal (*N* = 17)	11/13 (85%)	2/4 (50%)	11/13 (85%)	2/4 (50%)	5.5
Extra-temporal (*N* = 13)	4/6 (67%)	3/7 (43%)	4/8 (50%)	3/5 (60%)	1.5
ESI with spikes (*N* = 23)					
Temporal (*N* = 15)	9/11 (82%)	3/4 (75%)	9/10 (90%)	3/5 (60%)	13.5
Extra-temporal (*N* = 8)	3/6 (50%)	1/2 (50%)	3/4 (75%)	1/4 (25%)	1.0
Morphometry (*N* = 27)					
Temporal (*N* = 17)	0/0	5/17 (29%)	0/12	5/5 (100%)	0.4
Extra-temporal (*N* = 10)	3/4 (75%)	2/6 (33%)	3/7 (43%)	2/3 (67%)	1.5

None of the patients presented congruent results of all four types of imaging (PET, SPECT, ESI, MAP) so we limited our analysis to combinations of 2 and 3 exams. We did not find a relationship between number of concordant exams and surgical outcome (*p* = 0.34).

None of the individual tests obtained an accuracy of > 60%, except ESI if only patients with IEDs were taken into consideration for statistical analysis (63%).

When combining two exams (and considering only patients with IEDs), all combinations with ESI scored higher than 60%, except ESI + MAP. Best results were obtained when ictal SPECT could be performed during evaluation and SISCOM was considered together with PET and ESI, with a sensitivity of 100% and an accuracy of 75%. If these 3 exams were concordant and the corresponding overlapping area was resected, the odds ratio (OR) to become free from disabling seizures was 11.

**Fig. 2:**
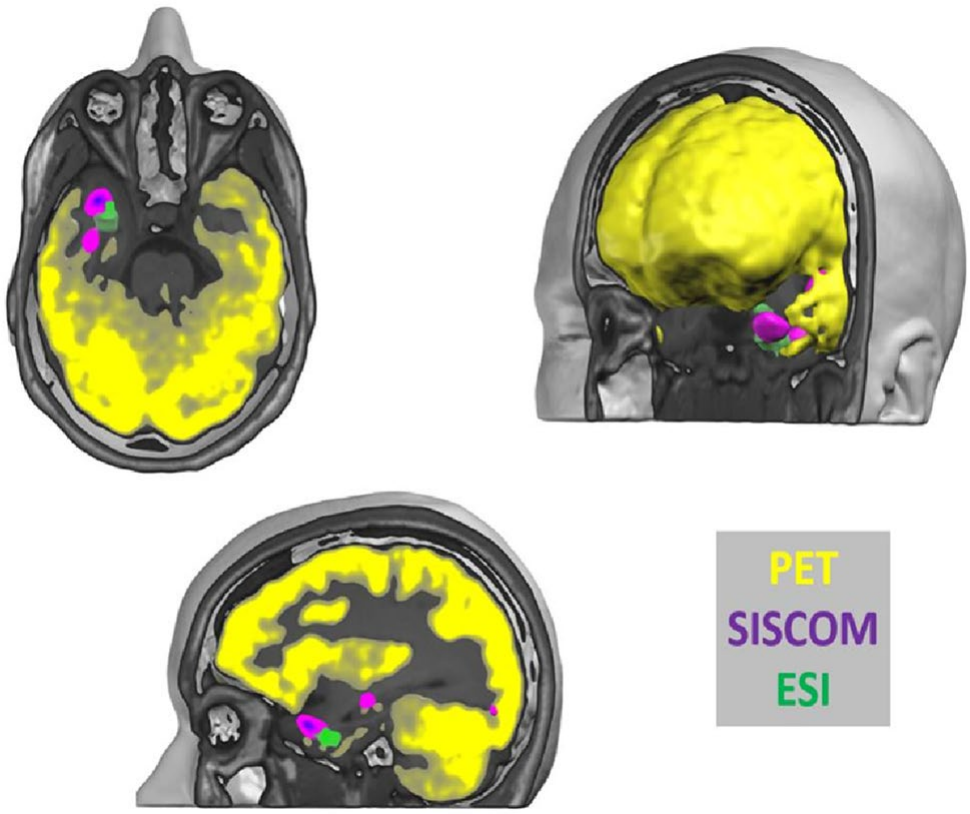
Female patient, 40 years with epilepsy since age of 14 y. All three imaging modalities (PET + SISCOM + ESI) were concordant for the left temporal pole, confirmed by phase II with depth electrodes. Morphometry was not carried out in this patient, due to lack of access at that time. Left temporal polectomy with amygdalo-hippocampectomy was carried out and she was seizure-free (follow-up 5 years)

**Fig. 3:**
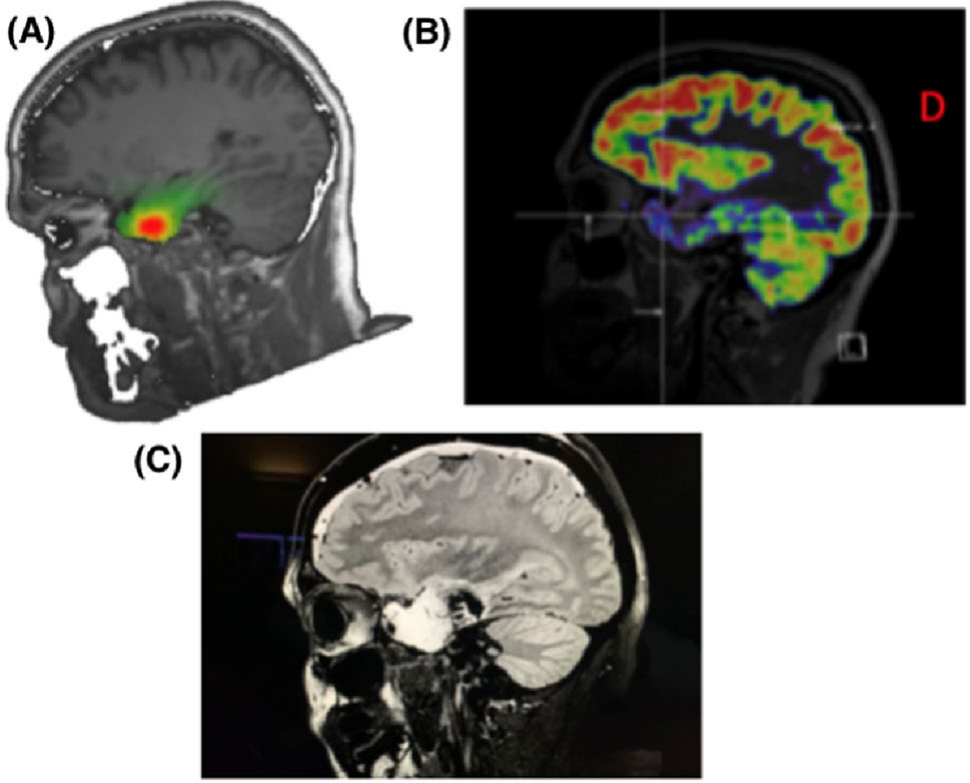
36 years old patient, epilepsy since the age of 19 years. Presurgical evaluation showed concordance of ESI (**A**) and PET (**B**) with respect to a focus in the right mesial temporal lobe and temporal pole. SISCOM was not performed in this patient. No lesion was depicted by morphometry. Phase II was performed, indicating seizure onset in the right mesial and lateral temporal cortex. An anterior temporal lobectomy was performed, with no clinical improvement after 2 years

**Fig. 4:**
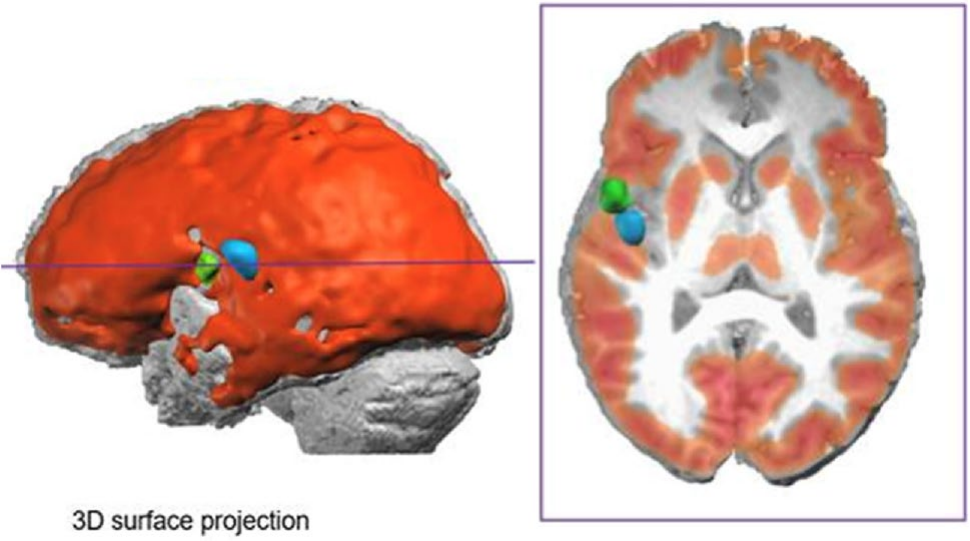
This 19 years old female ETLE patient was known for seizures since the age of 4. All three imaging modalities (PET, SPECT, ESI) were concordant for the insular region and the frontal and temporal opercular region. She was operated at the age of 7 years and resection resulted in an excellent outcome (ILAE I) after 12 years of follow-up. No phase II was carried out prior to surgery, due to complete concordance of the results. Blue: maximum perfusion of ictal SPECT, green: ESI, red: PET metabolism is found in red with an area of absent concordant with ESI and ictal SPECT. Left is left in this and all figures

A successful illustrative case of full correlation of PET, SPECT, and ESI is shown in Figs. 2 and 4. A more complicated case is shown in Fig. 3.

If we compare the yield for temporal and extratemporal lobe epilepsy separately, PET, SISCOM, and ESI performed equally well in TLE and ETLE, despite a tendency for ESI to perform better in TLE. Morphometric MRI analysis did not provide any positive result in TLE patients, so its sensitivity is 0 vs 75% in ETLE (*p* < 0.001).

Regarding histopathology, in 7 individuals type 2 focal cortical dysplasia was identified. All had an ILAE 1–2 post-operative outcome. In the remaining 26 patients, significantly less of them had a good outcome [15 (58%); *p* = 0.03].

## Discussion

In this single-center retrospective study, we evaluated the surgical outcome of non-lesional focal epilepsy, as determined by an expert neuroradiologist, after a mean follow-up of 5 years. Two main findings emerged: (a) despite the absence of an epileptogenic lesion by visual MRI analysis, the chances of being free from seizures with alteration of consciousness are 70%, with comparable success rate in both patients with TLE and ETLE; (b) the rigorous use of nuclear and electrical source imaging as well as morphometric MRI analysis is associated with an outcome almost as high as in lesional epilepsy.

In a multicenter retrospective study from 16 European centers, 108 non-lesional temporal lobe epilepsy (TLE) but only 40 extratemporal lobe epilepsy (ETLE) patients underwent surgery during the considered 4-year period, with improved outcome for TLE only in 2012–2013, compared to 15 years earlier [24]. While non-lesional TLE has benefited from progress in diagnostic tools (seizure-free rates rose from 48 to 68%), outcome of non-lesional ETLE remained poor with 30% in 1997–1998 and 37% in 2012–2013. Despite the increased use of complementary imaging tools in 2012–2013, full access to all techniques was not provided or not requested in most centers and its use was discussed on a case-by-case basis (e.g., for PET), often due to limitations imposed by the insurance provider. This may result in imprecise focus localization and imprecise electrode positioning during phase 2 evaluations, or worse, depriving patients of invasive monitoring and possible curative surgical treatment.

Regarding nuclear imaging, many centers do not have in-house facilities and therefore have to send their patients to other hospitals for cerebral FDG-PET. In most of the studies, EEG-monitoring before, during, and after tracer injection is not performed, so subclinical seizures may go undetected, leading rather to hyper- than hypometabolism and false-negative findings [25, 26]. In addition, the time of the last seizure may not be actively asked, incorrectly, or unreported by the patient. FDG-PET has been reported to be particularly useful in temporal lobe epilepsy with normal MRI [27, 28], if FDG-PET was unilateral. [27, 29–31]. Noncongruent FDG-PET or bilateral hypometabolism was associated with the poorest outcome (Engel class III–IV) [27, 30, 32].

Ictal SPECT requires constant surveillance of both the patient and EEG with trained personnel, to obtain a truly ictal exam, as well as permission by national authorities to perform injections with radioactive material. Consequently, not all centers can offer this exam. The contribution of SISCOM in non-lesional epilepsy remains controversial. Several studies found a positive association of focal SISCOM changes if the area of maximal hyperperfusion was included as part of the resection [33, 34]. Other studies suggested that the contribution of SISCOM was less significant for the surgical outcome in patients with non-lesional extra-temporal epilepsy [5]. In the present study, its yield was comparable to PET and the contribution of SISCOM appears to be complementary. While with PET, deep foci are more difficult to visualize due to an exponential decrease of signal with distance from the surface, SISCOM is more robust than PET in this respect, but also reflects seizure propagation and not necessarily seizure onset. Moreover, PET could appear pseudonormal if increased epileptic activity leads to hypermetabolism in a hypometabolic area, whereas ictal SPECT is independent of chronic subtle seizure activity.

ESI is an imaging tool of the underlying epileptogenic activity, like SISCOM, and has shown its excellent accuracy in a number of prospective and retrospective studies [20, 35–37]. If a lesional MRI and ESI are concordant and resected, the chances to benefit from surgery are excellent (OR 11; [38]). ESI in non-lesional epilepsy is promising as well, as suggested by a small previous observation from our center on 10 patients with normal MRI [39]. Interestingly, in a larger study on non-lesional extratemporal lobe epilepsy, interictal EEG was the best localizing exam with respect to post-operative seizure control [5]. However, like SISCOM, ESI may occasionally localize propagation than the seizure onset zone as suggested by Case No 2 (Fig. 3).

Morphometric MRI analysis (MAP) is particularly interesting in case of a positive result and leads to a 90% seizure-free outcome if the detected region was fully resected by surgery [40]. The main limitation of MAP remains in its restricted focus. While the other diagnostic tools (PET, SPECT, and ESI) screen for evidence of epileptogenic pathology in general, morphometric analysis aims at detecting a certain type of pathology (i.e., focal cortical dysplasia in the neocortex) and is limited to this task. This explains why MAP is unrevealing when the pathology is in the hippocampal or amygdala area or only consists of discrete gliosis. Accordingly, in the present study, the sensitivity of MAP for patients with TLE was 0%, as the vast majority of them had pathology in the temporo-mesial structures that was not dysplasia. Furthermore, it appears that this method is particularly sensitive to dysplasia type IIb, but less for type IIa, which is also difficult to identify visually. However, the predictive value is very high, i.e., if the algorithm identifies an anomaly; it is associated in more than 90% with the epileptogenic zone. Furthermore, in the ETLE group, where the prevalence of FCD is higher, MAP achieved a sensitivity of 75% in this study, and this is based on MRIs radiologically assessed as unremarkable. Extra-temporal non-lesional patients are among the most complicated to treat and, in the present study, MAP has shown its utility in particular in this group.

Our main result resides in the significant additional yield of the combination of exams, coupled to co-registration of all data sets within the patient’s individual MRI reference volume. We obtained a very high OR of 11 if PET, SISCOM, and ESI were combined and concordant. None of our patients was concordant for all four exams, but it appears logical that such a concordance should be related to an ever higher chance of post-operative seizure control.

Many studies underlined the need for high-resolution MRI and, to a somewhat lesser degree, the need for full scalp electrode coverage during evaluation [20]. ESI (or for magnetoencephalogram, MSI) is not adopted in all centers, although analysis can be outsourced, provides a high accuracy of 75–78% with 25–38 electrodes, and does not require anymore a larger team of in-house specialists [20, 37] The combined analysis of MEG with other non-invasive imaging techniques was described in a recent study on 39 MRI-negative patients, with 69% seizure free at 1 year follow-up [41]. The high direct and indirect costs of PET and SISCOM make it less attractive to incorporate both in the center routine, although there is evidence from several observations, including our own, that more and more cases are non-lesional [42]. There is a turning point in the composition of referrals since 5–10 years: the increase of non-lesional cases and the relative “disappearance” of patients with hippocampal sclerosis, which requires a much more comprehensive infrastructure, beyond high-resolution MR imaging and video-EEG monitoring as suggested by a review of centers in the US, Australia, and Europe [24, 43]. In all our patients, lesions were not identified by a dedicated and experienced neuroradiologist.

Our study had several limitations, including its retrospective nature. This nevertheless helped to identify all patients and, based on the review of imaging results with resection volume and surgical outcome, to identify the optimal combination of exams. In our center, we offer all non-invasive imaging tools to patients with unrevealing MRI, which are accessible in-house (PET, SISCOM, ESI) or are outsourced (ESI, morphometry). Intracranial EEG was performed in 28/33 (85%) of patients which could not be obviated by our presurgical protocol. However, the application of the full battery of non-invasive imaging allowed optimal determination of the implantation plan and eventually surgical strategy, leading to successful surgical therapy.

With increasing experience over the years, we developed an efficient protocol allowing carrying out a comprehensive presurgical evaluation on average within 10 days. Nevertheless, the overall number of ultimately operated patients with non-lesional epilepsy is relatively small, reflecting the cautious attitude of the treating physicians and of the patient: based on earlier meta-analyses, very low odds of surgical success are given during the pretreatment consultation. This discourages patients from pursuing epilepsy surgery, similar to observations elsewhere [42] although the latest studies, including the present study, report around 60–70% likelihood of control of disabling seizures in carefully selected patients after comprehensive work-up. We feel that a pessimistic view is no more justified, if the different tools to localize the focus are rigorously applied.

### Supplementary Information

Below is the link to the electronic supplementary material.

Supplementary file1 (DOCX 32 KB)

## Data Availability

The data that support the findings of this study are available from the corresponding author, [C.C., czarnetzkichris@gmail.com], upon reasonable request.
