# Supplementary material for: Yield of non-invasive imaging in MRI-negative focal epilepsy
Source: J Neurol. 2023 Nov 1;271(2):995–1003. doi: 10.1007/s00415-023-11987-6 (PMC10827933; doi:10.1007/s00415-023-11987-6)
Supplement: Supplementary file 1 — Supplementary file1 (DOCX 32 KB) [file 415_2023_11987_MOESM1_ESM.docx]

**Supplementary material**

**Tables**

**Table 2 – Results of individual patients**

| **Patient no.** | **Age Onset (years)** | **Op. age (years)** | **Gender** | **Operated lobe** | **Engel class** | **PET** | **Siscom** | **ESI** | **Morphometry** | **Phase 2** | **Histopathology** |
| --- | --- | --- | --- | --- | --- | --- | --- | --- | --- | --- | --- |
| **1** | 4 | 7 | F | Frontal and temporal opercular resection with left insular cortectomy. | 1 | Left Insula | Left insula | Left frontal temporal junction | Test not performed | No | Gliosis and ectopic neurons |
| **2** | 26 | 32 | M | Right medial post-central gyrus. | 1 | normal | Precentral gyrus (bilateral) | No spikes | Precentral gyrus | Yes | Focal cortical dysplasia type Ia |
| **3** | 3 | 10 | M | Right parietal opercular resection. | 2 | normal | Right operculum and posterior Insula | Right operculum and posterior insula | Right operculum and Insula | Yes | Ectopic neurons |
| **4** | 2 | 10 | F | Frontal left cortectomy. | 1 | Frontal left | Frontal left > Frontal right | No spikes | Left central region | Yes | Focal cortical dysplasia type IIb |
| **5** | 7 | 55 | F | Left anterior cingular gyrus resection. | 1 | normal | normal | No spikes | Left cingulate gyrus | Yes | Focal cortical dysplasia type IIb |
| **6** | 0.5 | 16 | M | Frontal and parietal opercular right cortectomy. | 3 | Frontal right operculum | Frontal right operculum | No spikes | Test not performed | Yes | Gliosis |
| **7** | 3.5 | 9 | F | Parietal left cortecomy. | 4 | normal | Left Putamen | Temporal | Test not performed | Yes | Gliosis |
| **8** | 1 | 35 | F | Frontal right lobectomy. | 1 | normal | Right cingular lobe + frontal polar | Frontal Right parasagittal | normal | Yes | Focal cortical dysplasia IIa |
| **9** | 15 | 18 | F | Left frontal anterior cortectomy. | 2 | normal | Frontal left | Frontal left | normal | Yes | Gliosis |
| **10** | 19 | 21 | F | Right orbitofrontal and frontopolar cortectomy. | 2 | Temporal mesial Right + Right orbitofrontal | Right Temporal lobe | Right Frontal lobe | normal | Yes | Ectopic neurons |
| **11** | 13 | 27 | F | Left Parietal opercular resection. | 1 | Left temporal pole + mesial temporal lobe + Left parieto-insular. | Temporal mésial G < central D + Parietal Left | Left temporal pole | normal | Yes | Focal cortical dysplasia type IIb |
| **12** | 10 | 30 | F | Left frontal opercular and inferior part of precentral gyrus resection | 1 | Left Precentral gyurs | Bilateral multifocal | No spikes | normal | Yes | Gliosis |
| **13** | 11 | 20 | F | Posterior temporo-parietal junction | 1 | normal | Posterior portion of left temporal lobe | Temporal left posterior | Posterior temporo-parietal junction | Yes | Focal cortical dysplasia type IIa |
| **14** | 3 | 33 | F | Right temporal pole resection and amygdalo-hippocampectomy | 1 | normal | Right temporal lobe | Right mesial temporal lobe | normal | Yes | Gliosis |
| **15** | 8 | 18 | M | Left temporal pole and mesial temporal lobe resection. | 2 | Temporal left | Left lateral temporal lobe + Left insula | Left lateral temporallobe | normal | Yes | Ectopic neurons and gliosis |
| **16** | 21 | 29 | F | Right temporal pole and mesial temporal lobe resection. | 3 | Right temporal lobe | Right temporal lobe + right Insula | Temporal right + Right insula | normal | Yes | Gliosis and ectopic neurons |
| **17** | 13 | 26 | F | Left temporal pole resection and amygdalohippocampectomy | 1 | Temporal left | Temporal left | Temporal left | normal | Yes | Gliosis |
| **18** | 5 | 19 | F | Left mesial temporal lobe resection | 1 | Frontal-polar Right | Orbitofrontal right and temporal right | Orbitofrontal right and temporal right | normal | Yes | Gliosis and ectopic neurons |
| **19** | 14 | 35 | M | Left temporal pole and amygdala resection (sparing hippocampus) | 4 | Temporal left | Temporal left | Temporo-occiptal left (multifocal) | normal | Yes | NA |
| **20** | 15 | 33 | M | Left temporal lobectomy and amygdalohippocampectomy | 1 | Temporo-parietal left | Fronto-temporal left (multifocal) | Temporal left | Test not performed | Yes | NA |
| **21** | 16 | 39 | F | Right temporal pole and hippocampectomy (sparing tail of hippocampus) | 1 | Bilateral frontoparietal lobe | Temporal right and insula right | Temporal Right | Test not perfomed | Yes | Gliosis and ectopic neurons |
| **22** | 5 | 7 | M | Temporal left cortectomy | 1 | Temporal left | Temporal left | Temporal left | Test not performed | No | Focal cortical dysplasia |
| **23** | 0.3 | 11 | M | Superior temporal gyrus resection | 1 | normal | Test not performed | No spikes | normal | Yes | Gliosis |
| **24** | 13 | 14 | M | Temporal left cortectomy | 1 | Temporal left | Test not performed | Temporal left | normal | Yes | Gliosis |
| **25** | 4 | 49 | F | Temporal left (sparing mesial temporal structures) + temporal left polectomy | 1 | Temporal right | Temporal right | Temporal right | normal | No | Gliosis and ectopic neurons |
| **26** | 23 | 36 | M | Right superior and middle temporal gyrus resection (sparing mesial temporal structures) | 4 | Normal | Bitemporal | Bitemporal | normal | Yes | Normal |
| **27** | 0.5 | 27 | M | Left temporal pole + amygdalohippocampectomy | 1 | Temporal left | Temporal left | No spikes | normal | Yes | Normal |
| **28** | 28 | 48 | M | Superior temporal gyrus and mesial temporal structures resection | 1 | Temporal right | Bitemporal | No spikes | normal | Yes | Gliosis and ectopic neurons |
| **29** | 22 | 35 | F | Temporal pole resection and amygdalohippocampectomy | 1 | Temporal left | Temporal left and insular left | No spikes | normal | Yes | Normal |
| **30** | 12 | 43 | F | Right temporal, mesial temporal structures and insula resection | 1 | Temporal right + Insular right | Temporal right + Insular right + Frontal right | Temporal right and insular right | normal | Yes | Normal |
| **31** | 20 | 37 | M | Lobectomy + amygdalohippocampectomy | 4 | Temporal right + opercular right | Test not performed | Temporal right | normal | Yes | Normal |
| **32** | 14 | 21 | M | Temporal lobectomy + amygdalohippocampectomy | 1 | Temporal right mesial | Temporal right mesial | No spikes | normal | No | Gliosis |
| **33** | 21 | 48 | M | Right temporo-parieto-occipital junction removal | 1 | normal | Temporol right | Temporal right | normal | Yes | Normal |

NA: no abnormalities
